# Motor Outcomes of Robot-Assisted Versus Conventional Occupational Therapy for Upper-Limb Recovery in Subacute Stroke: A Retrospective Cohort Study with Exploratory Neurocognitive Outcomes

**DOI:** 10.3390/jcm15093512

**Published:** 2026-05-04

**Authors:** Eunju Na, Sumin Lee, Joon Won Seo, Seung Ok Nam, Eunyoung Kang, Dong-Hyuk Kim, Sunghoon Lee, Soo-Hyun Soh, Hyung-Ju Na, Younkyung Cho

**Affiliations:** 1 Department of Rehabilitation Medicine, Kwangju Christian Hospital, Gwangju 61661, Republic of Korea; naunju@naver.com (E.N.); leesm681@naver.com (S.L.); jwseokorea@gmail.com (J.W.S.); vienna0228@naver.com (S.O.N.); eykang74@hanmail.net (E.K.); north317@daum.net (D.-H.K.); starhoon3@hanmail.net (S.L.); skylove422@gmail.com (S.-H.S.); 2 Seoul-Metropolitan Dongbu Hospital, Seoul 02534, Republic of Korea; ronaljuu@naver.com

**Keywords:** stroke rehabilitation, robot-assisted therapy, upper-limb rehabilitation, motor recovery, cognitive impairment, activities of daily living

## Abstract

**Background/Objectives**: Robot-assisted therapy (RAT) can deliver repetitive, feedback-enriched upper-limb practice after stroke, but evidence comparing RAT with dose-matched conventional occupational therapy (COT) under routine inpatient conditions—and concurrent neurocognitive data—remains limited. We compared motor recovery between an end-effector RAT-based program (30 min RAT plus 30 min COT) and dose-matched COT alone in subacute stroke survivors, with neurocognitive outcomes prespecified as exploratory endpoints. **Methods**: In this single-center retrospective non-randomized cohort study, adults with first-ever ischemic or hemorrhagic stroke who completed routine baseline and week−4 assessments received 4 weeks of upper-limb rehabilitation: combined RAT plus COT (60 min daily) or COT alone (60 min daily). The primary outcome was the week-4 Fugl–Meyer Assessment–Upper Extremity (FMA-UE) motor score adjusted for baseline. Primary inference used covariate-adjusted linear regression on outcome-specific complete cases, with a prespecified stabilized inverse probability of treatment weighting (IPTW) average treatment effect analysis as the sensitivity test. Secondary and exploratory endpoints were interpreted descriptively; Benjamini–Hochberg false discovery rate (FDR) control and multiple imputation were applied as supportive analyses. **Results**: The analytic cohort comprised 65 patients (RAT, *n* = 33; COT alone, *n* = 32). Both groups improved over 4 weeks, but the RAT group had worse baseline upper-limb motor status. The adjusted between-group difference for the week-4 FMA-UE motor score was non-significant (adjusted mean difference, 4.39; 95% confidence interval (CI), −2.43 to 11.21; *p* = 0.203), and the stabilized IPTW estimate was concordant (β = 2.17; 95% CI, −3.63 to 7.98; *p* = 0.464). In unadjusted analyses, the FMA-UE motor gain was larger after RAT than COT alone (14.70 ± 15.53 vs. 7.91 ± 9.42), and only the RAT group exceeded the prespecified 12.4-point clinically important threshold; this signal attenuated after adjustment. No secondary or exploratory endpoint remained significant after FDR control. Multiple imputation for the primary endpoint was concordant with the complete-case result (pooled β = 4.52; 95% CI, −1.91 to 10.94; *p* = 0.168). **Conclusions**: End-effector RAT did not demonstrate adjusted superiority over dose-matched COT alone for upper-limb motor recovery. The larger unadjusted FMA-UE gain should be interpreted as a descriptive impairment-level signal rather than as evidence of comparative efficacy. Neurocognitive results were exploratory; the retrospective non-randomized design, baseline imbalance, differential missingness, and unavailable confounder data require cautious interpretation.

## 1. Introduction

Stroke remains a leading cause of long-term disability worldwide, and its global burden continues to grow despite advances in acute management and secondary prevention [1]. Among post-stroke sequelae, upper-limb dysfunction is particularly disabling because it directly limits self-care, daily activity performance, and long-term independence [2]. Recovery of arm and hand function is often incomplete—especially in patients with severe early paresis—and residual upper-limb deficits remain a major barrier to functional reintegration [3]. Cognitive impairment frequently coexists after stroke and is strongly linked to reduced functional outcome, diminished quality of life, and increased risk of future dementia [4]. Deficits in attention, executive function, and visuospatial processing can interfere with task engagement, motor learning, and carryover during therapy, thereby constraining upper-limb recovery [5,6]. Together, these observations argue for rehabilitation approaches that are intensive and task-oriented while remaining interpretable within a broader cognitive context.

Robot-assisted therapy (RAT) has gained attention because it can operationalize key principles of motor learning and experience-dependent neuroplasticity in a reproducible manner. Effective neurorehabilitation depends on practice that is use-dependent, task-specific, repetitive, sufficiently intensive, salient, and progressively challenging [7,8]. Robotic platforms are well-suited to provide these conditions through structured movement repetition, standardized assistance, graded task difficulty, and real-time multimodal feedback [8]. Among robotic systems, end-effector devices are particularly relevant to routine inpatient practice because they are practical to implement and can deliver high-volume, feedback-enriched reaching training, although robotic platforms are not clinically interchangeable [9,10].

Existing evidence suggests that RAT can improve upper-limb impairment after stroke, although effects on activity-level outcomes are heterogeneous and appear to depend on device characteristics, stroke phase, treatment dose, and comparator intensity [11,12,13,14]. When RAT is compared with active, dose-matched conventional therapy rather than lower-intensity controls, clear superiority has been difficult to demonstrate [11,12,13,14]. Real-world comparisons between RAT and dose-matched conventional occupational therapy (COT) remain scarce, particularly in subacute stroke populations treated under routine inpatient conditions [13,14,15].

Cognition is an additional unresolved issue in the robotic rehabilitation literature. Cognitive status has often been used as an exclusion criterion rather than examined as a co-determinant or measurable dimension of recovery. A systematic review by Bressi et al. found that formal cognitive outcome assessment in robotic upper-limb studies was uncommon and methodologically heterogeneous [16]. More broadly, rehabilitation trials frequently underrepresent medically complex inpatients commonly encountered in daily clinical practice [17]. This is relevant because cognitive reserve and frailty may act not only as background characteristics, but also as clinically meaningful confounders of motor recovery. Cognitive reserve refers to the resilience conferred by lifetime intellectual, educational, occupational, and social experience; recent systematic reviews suggest that higher reserve is associated with better post-stroke cognitive and functional recovery [18]. Frailty reflects reduced physiological reserve and multisystem vulnerability; contemporary systematic review and cohort data indicate that frailty is associated with poorer functional outcome after stroke and less favorable rehabilitation trajectories among older inpatients [19,20]. Because RAT requires sustained attention, task engagement, visuomotor processing, and tolerance of high-dose practice, between-patient differences in reserve and frailty may plausibly modify responsiveness to robotic rehabilitation in pragmatic clinical cohorts. A real-world comparison that acknowledges these patient-level modifiers is therefore clinically relevant, even when such variables cannot be fully measured in retrospective datasets.

Against this background, even cognition-oriented robotic rehabilitation studies have generally enrolled patients with limited cognitive impairment and have often reported motor outcomes comparable—rather than clearly superior—to dose-matched conventional therapy [10]. We therefore conducted a real-world cohort comparison of a combined RAT plus COT program and dose-matched COT alone for upper-limb recovery in subacute stroke. Motor recovery was designated as the primary focus, and neurocognitive outcomes were prespecified as exploratory and hypothesis-generating. We hypothesized that RAT would yield a clinically meaningful signal in upper-limb motor recovery while also providing pragmatic data on the neurocognitive profile of recovery under routine clinical conditions.

## 2. Materials and Methods

### 2.1. Study Design and Participants

This single-center retrospective non-randomized comparative cohort study was conducted at the Department of Rehabilitation Medicine, Kwangju Christian Hospital, Republic of Korea. Clinical data were retrieved from electronic medical records spanning December 2023 through December 2024, and reporting followed the Strengthening the Reporting of Observational Studies in Epidemiology (STROBE) guidelines. Because treatment assignment was not randomized, each patient was analyzed according to the rehabilitation program delivered during routine inpatient care.

The four-week follow-up timepoint corresponded to the hospital’s routine inpatient reassessment schedule after approximately 20 treatment days of upper-limb rehabilitation. In this real-world cohort, week 4 was considered an appropriate pragmatic timepoint for capturing short-term impairment-level change during the early subacute rehabilitation phase while limiting variability related to discharge timing. This choice was consistent with previous upper-limb robotic rehabilitation studies in subacute stroke that used 3- to 4-week intervention blocks or week-4 post-treatment assessments [2,21,22,23]. We recognize, however, that recovery in older stroke survivors can be more protracted and that the broader subacute neuroplastic window extends beyond 4 weeks; the present study was therefore designed to evaluate early treatment response rather than definitive longer-term recovery.

Patients were eligible if they had sustained a first-ever ischemic or hemorrhagic stroke verified by brain computed tomography or magnetic resonance imaging and had been admitted or transferred for rehabilitation within 6 months of stroke onset. Exclusion criteria comprised onset more than 6 months before rehabilitation enrollment, traumatic brain injury, monoparesis, tetraplegia, absence of motor weakness, prior stroke, other neurologic conditions that could confound outcome assessment, and failure to complete a baseline or 4-week follow-up assessment for any prespecified outcome. The analytic cohort was thus limited to patients who completed both baseline and week-4 assessments for at least one prespecified outcome.

Because the study was retrospective, the exact treatment-allocation algorithm could not be fully reconstructed. Group assignment to RAT or COT arose from routine clinical judgment and service logistics rather than a protocolized random process and likely reflected baseline motor severity, perceived ability to engage with robotic training, therapist or physiatrist preference, and device availability.

### 2.2. Interventions

Both groups followed a dose-matched upper-limb rehabilitation program over 4 weeks, delivered 5 days per week for 60 min per day. Each daily session consisted of two 30-min blocks, totaling 40 sessions across 20 treatment days. Total daily treatment time was identical between groups. The present comparison should therefore be understood as a combined-program contrast—RAT versus COT—rather than as a comparison of robot-only treatment versus conventional occupational therapy.

#### 2.2.1. Robot-Assisted Training Group

Patients in the RAT group received 30 min of robot-assisted upper-limb training on the Rebless Planar^®^ (H-ROBOTICS Inc., Incheon, Republic of Korea), followed by 30 min of conventional occupational therapy each treatment day. The conventional block followed the same institutional COT framework used in the COT group (see below). Robotic sessions consisted of repetitive, task-oriented reaching exercises in the horizontal plane and were delivered in passive, active-assistive, or active mode according to motor capacity and fatigue. Visual feedback was displayed in real-time throughout each session, and the treating therapist adjusted task difficulty based on individual performance.

#### 2.2.2. Conventional Occupational Therapy Group

Patients in the COT group received 60 min of conventional occupational therapy daily, divided into two 30-min sessions delivered by licensed occupational therapists. Treatment followed a structured COT framework that incorporated neurodevelopmental treatment principles, passive and active range-of-motion exercises, strengthening activities, facilitation of voluntary movement, and task-oriented upper-limb training—including grasp-and-release practice and object manipulation—aimed at promoting functional use of the paretic arm in activities of daily living (ADL).

#### 2.2.3. Standardization and Fidelity of Conventional Occupational Therapy

COT was delivered according to a shared institutional framework intended to improve reproducibility across therapists and groups. In general, each 30-min COT block emphasized active therapist-guided upper-limb practice and typically included: (1) preparatory positioning and scapular/shoulder mobilization with passive or active-assisted range-of-motion as needed; (2) facilitation of selective voluntary movement and proximal postural control; (3) progressive practice of reaching, grasping, release, forearm rotation, and object manipulation; and (4) task-oriented training linked to basic activities of daily living, such as cup handling, grooming-related reaching, dressing components, bilateral support tasks, and transfer-related upper-limb use [2,24].

Within this framework, therapists individualized task selection and progression according to each patient’s motor severity, fatigue, pain, tone, cognition or communication status, functional goals, and treatment tolerance. Difficulty was graded by adjusting therapist assistance, repetition count, movement amplitude, object size or weight, task distance and height, and postural demands; the exact mix of exercises and ADL-oriented tasks therefore varied between patients while preserving the same overall protocol structure.

Because this study was retrospective, prospective fidelity checklists and minute-by-minute repetition counts were not available for all sessions, and inter-therapist reliability was not quantitatively measured. Inter-therapist consistency was nonetheless promoted by a fixed daily treatment dose, delivery by licensed occupational therapists within a single department, and use of a common task-oriented treatment framework.

### 2.3. Robotic Device

**Figure 1:**
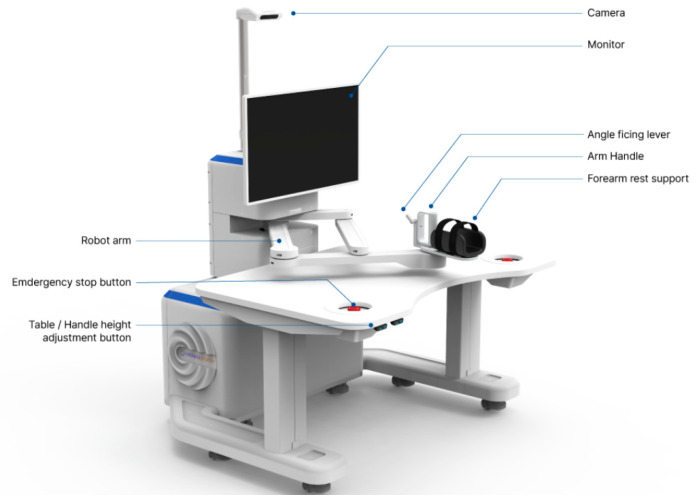
Configuration of the Rebless Planar^®^ (H-ROBOTICS Inc., Incheon, Republic of Korea).

**Figure 2:**
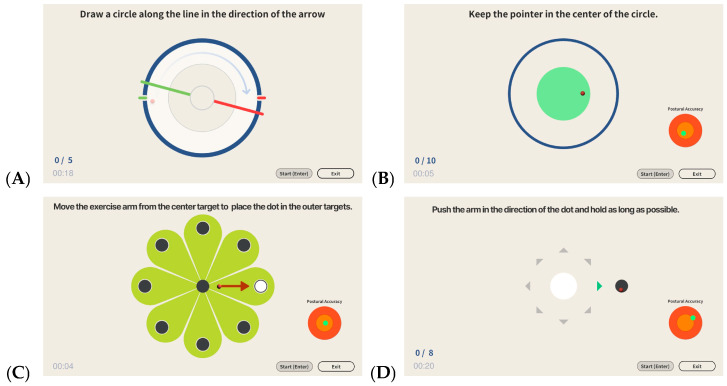
Four rehabilitation programs on the Rebless Planar^®^ (H-ROBOTICS Inc., Incheon, Republic of Korea). (**A**) Circle; (**B**) Round dynamic; (**C**) Point-to-Point; (**D**) Playback static. (**A**) Circle: patients trace the displayed circular guide path, and the arrow/triangular marker indicates the instructed movement direction. (**B**) Round dynamic: patients maintain the handle/pointer within the central circular target against resistance. (**C**) Point-to-Point: patients move from the central starting point toward peripheral circular targets, with the colored sectors indicating target zones. (**D**) Playback static: patients push the handle toward the displayed target dot and hold the position. The arrows/triangular markers, colored areas, circles/dots, and guide lines are software-generated visual guidance cues indicating movement direction, target location, holding zone, or prescribed trajectory; these graphical elements were not analyzed as separate study variables.

Robot-assisted training was performed using the Rebless Planar^®^ (H-ROBOTICS Inc., Incheon, Republic of Korea), an end-effector rehabilitation robot designed for planar reaching movements (Figure 1). The device supports passive, active-assistive, and active training modes and provides real-time visual feedback together with game-based exercise programs (Figure 2), in keeping with the goal of repetitive, goal-directed, feedback-enriched practice after stroke.

### 2.4. Outcome Measures

All outcomes were derived from routine clinical assessments performed at baseline and at 4 weeks. The primary outcome was the Fugl-Meyer Assessment-Upper Extremity (FMA-UE) motor score. Secondary motor and functional endpoints were the Fugl-Meyer Assessment (FMA) total score, Manual Function Test (MFT), grip strength, global functional status, and activities of daily living; neurocognitive measures were prespecified as exploratory. Analyses used observed data without imputation, and each endpoint included only participants with both baseline and week-4 assessments for that outcome.

#### 2.4.1. Upper-Limb Motor and Manual Function

Upper-limb motor impairment was assessed with the FMA-UE motor scale (score range 0–66). Manual performance was evaluated with the MFT. Grip strength of the affected hand was recorded in pounds (lb).

#### 2.4.2. Global Function and Activities of Daily Living

Global functional status and independence in daily activities were assessed with the Functional Independence Measure (FIM) and the Korean Modified Barthel Index (K-MBI).

#### 2.4.3. Neurocognitive Assessment

For reporting purposes, exploratory neurocognitive outcomes were organized into four conceptual domains: language (Boston Naming Test; Language Comprehension and Repetition Test), executive function (Stroop Color, Word, and Color–Word subtests), visuospatial and constructional ability (Right–Left Orientation Test; Stick Construction and Visual Recognition Test), and multidomain screening (Clock Drawing Test). These groupings were intended as a descriptive framework and were not treated as confirmatory domain-level endpoints.

### 2.5. Statistical Analysis

All analyses were performed using IBM SPSS Statistics for Windows, version 27.0 (IBM Corp., Armonk, NY, USA). Continuous variables are presented as mean ± standard deviation and categorical variables as count (%). Normality was assessed with the Shapiro–Wilk test. Between-group baseline characteristics were compared using the independent *t*-test or Mann–Whitney U test for continuous variables and the chi-square or Fisher’s exact test for categorical variables, as appropriate. Within-group changes from baseline to week 4 were analyzed with the paired *t*-test or Wilcoxon signed-rank test. All tests were two-sided, with statistical significance set at *p* < 0.05.

The primary between-group analysis used covariate-adjusted linear regression with the week-4 score as the dependent variable. Treatment group, baseline score, age, sex, stroke type, onset duration, and baseline Mini-Mental State Examination (MMSE) were entered as covariates. The same adjusted framework was applied to secondary motor, functional, and exploratory neurocognitive outcomes as supportive analyses, given the non-randomized design and the baseline motor imbalance. Raw change scores (Δ = post − pre) were reported descriptively and were not interpreted as evidence of comparative efficacy.

A prespecified propensity-score-based sensitivity analysis was conducted for the primary outcome using stabilized inverse probability of treatment weighting (IPTW) for the average treatment effect (ATE). The propensity-score model included age, sex, stroke type, paretic side, baseline K-MBI, baseline MMSE, and baseline FMA-UE motor score, selected a priori as plausible determinants of treatment allocation and recovery. Covariate balance was assessed using standardized mean differences. Because IPTW addresses only measured covariates, this analysis was interpreted as a sensitivity test rather than as causal proof.

For the main responder analysis, a single FMA-UE threshold of 12.4 points was prespecified to avoid inflation from multiple published minimal clinically important difference (MCID) estimates and to reflect values applicable to moderate-to-severe upper-limb paresis [15,25]. Responder analyses for FIM, K-MBI, hand grip strength, and the Boston Naming Test were retained as supportive exploratory analyses. Results are presented as mean changes, adjusted mean differences, or odds ratios (ORs) with 95% confidence intervals (CIs) and *p* values.

Only the primary endpoint was treated as confirmatory. Secondary and exploratory endpoints were interpreted descriptively, and Benjamini–Hochberg false discovery rate (FDR) adjustment was applied to the family of nonprimary endpoints as a supportive sensitivity analysis [26]. Several clinically relevant candidate confounders—including lesion location and size, spatial neglect, aphasia severity, depression or mood status, nutritional status, polypharmacy, pre-stroke physical activity level, relevant medication exposure, and concurrent non-study therapies—were considered but were not collected prospectively as consistently coded, standardized, and temporally aligned baseline variables suitable for patient-level adjustment. They were therefore not included in the covariate-adjusted or propensity-score models, and their potential influence was addressed as residual confounding in the interpretation of the findings.

The extent and pattern of missing follow-up data were summarized by treatment group, and baseline characteristics were compared between patients with complete week-4 follow-up and those with missing follow-up. Because follow-up missingness was concentrated in the COT group, a supportive multiple-imputation by chained equations analysis was performed for the primary outcome under a missing-at-random assumption. The imputation model included treatment group, baseline FMA-UE motor score, age, sex, stroke type, onset duration, baseline MMSE, baseline K-MBI, and baseline FIM; 30 imputations were generated, and pooled estimates were obtained using Rubin’s rules [27,28].

Generative Artificial Intelligence (AI) Disclosure: Generative AI assistance was used only for English language editing and for drafting the initial participant flow diagram; no AI tools were used for the study design, data collection, statistical analysis, or interpretation of the findings.

## 3. Results

### 3.1. Study Population

**Figure 3:**
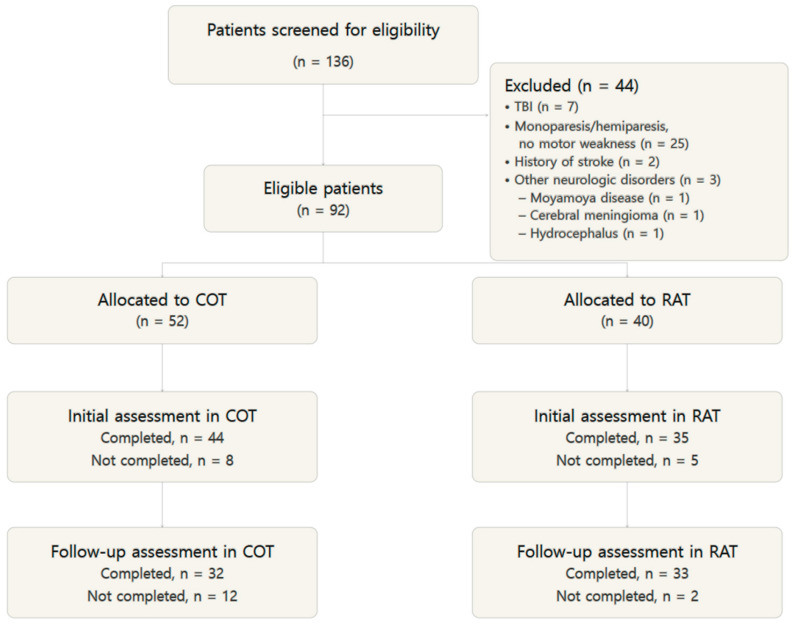
Flow diagram of participant screening, allocation, and outcome assessment. Abbreviations: COT, conventional occupational therapy; RAT, robot-assisted therapy; TBI, traumatic brain injury.

Of the 136 patients initially screened, 44 were excluded on the basis of predefined clinical criteria: traumatic brain injury (*n* = 7); monoparesis, tetraplegia, or no motor weakness (*n* = 25); prior stroke (*n* = 2); onset duration exceeding 6 months (*n* = 7); and other neurologic disorders (*n* = 3; Moyamoya disease, n = 1; tentorial meningioma, *n* = 1; hydrocephalus, *n* = 1). Among the 92 remaining eligible patients, 13 were excluded because no baseline assessment was completed for any prespecified outcome and 14 because no 4-week follow-up was available, leaving a final analytic cohort of 65 patients—33 in the RAT group and 32 in the COT group (Figure 3). Observed-outcome analyses were performed on an outcome-specific complete-case basis; a separate multiple-imputation sensitivity analysis was performed for the primary endpoint.

In the COT group, 52 patients were potentially eligible; 8 lacked a baseline assessment and 12 lacked a 4-week follow-up, leaving 32 patients for analysis. In the RAT group, 40 patients were potentially eligible; 5 lacked a baseline assessment and 2 lacked a 4-week follow-up, leaving 33 patients for analysis.

The extent and pattern of missing data are summarized in Table 1. Among patients with baseline data for at least one prespecified outcome, week-4 follow-up was unavailable in 12 out of 44 COT patients (27.3%) and 2 out of 35 RAT patients (5.7%), most commonly because of early discharge or early treatment termination. Compared with the complete-case cohort, patients with missing follow-up had shorter onset-to-baseline duration and better baseline motor and functional status—a pattern inconsistent with fully random missingness (Supplementary Table S1). Because missing follow-up was concentrated in COT and was associated with better baseline status, the complete-case analyses may have preferentially removed potentially better-prognosis COT patients, biasing unadjusted between-group comparisons in a direction that could make RAT appear more favorable.

### 3.2. Baseline Characteristics

The final analytic cohort of 65 patients comprised 33 RAT and 32 COT patients. Baseline demographic and stroke-related characteristics were broadly similar between groups, with no significant differences in age, sex, stroke type, paretic side, onset-to-baseline duration, K-MBI, MMSE, or baseline neurocognitive measures. A marked baseline motor imbalance was nonetheless evident: the RAT group had significantly lower hand grip strength (1.58 ± 4.83 vs. 19.78 ± 24.34 lb, *p* < 0.001), FMA-UE motor score (18.18 ± 20.45 vs. 36.06 ± 27.14, *p* = 0.004), FMA total score (64.03 ± 27.55 vs. 85.25 ± 34.50, *p* = 0.008), and MFT score (6.94 ± 8.30 vs. 14.62 ± 12.17, *p* = 0.004) than the COT group (Table 2). This pattern is consistent with non-random clinical allocation and could bias crude between-group comparisons. Adjusted regression and the prespecified propensity-based sensitivity analysis were therefore prioritized over unadjusted contrasts when interpreting comparative treatment effects.

### 3.3. Motor and Functional Outcomes

In the primary adjusted analysis summarized in Panel B, RAT did not demonstrate statistically significant superiority over COT for any motor or functional outcome after controlling for baseline score, age, sex, stroke type, onset duration, and baseline MMSE. For the primary endpoint, the adjusted mean difference in week-4 FMA-UE motor score was 4.39 points (95% CI, −2.43 to 11.21; *p* = 0.203). Adjusted differences for all secondary motor and functional outcomes were also non-significant, with model-level details presented in Supplementary Table S2.

As shown in Table 3, both groups showed significant within-group improvement from baseline to week 4 across all motor and functional measures. In the RAT group, K-MBI, hand grip strength, FMA-UE motor, FMA total, MFT, and FIM all improved over the intervention period. The COT group showed the same overall pattern, although the within-group improvement in hand grip strength was more modest than for the other outcomes.

In Table 4, the only nominally significant unadjusted between-group change-score difference was observed for FMA-UE motor gain, which was larger in the RAT group than in the COT group (mean difference, 6.79 points; 95% CI, 0.42 to 13.16; *p* = 0.037). Other unadjusted motor and functional change-score comparisons were numerically favorable but non-significant or comparable between groups. Because the FMA-UE motor change-score difference was not retained in the adjusted analysis, all unadjusted comparisons are reported descriptively and should not be interpreted as evidence of adjusted comparative superiority.

### 3.4. Neurocognitive Outcomes

Adjusted exploratory analyses did not reveal a consistent neurocognitive advantage for either group. The only nominally significant adjusted estimate favoring RAT was for the Stick Construction and Visual Recognition Test (adjusted mean difference, 1.98; 95% CI, 0.04 to 3.91; *p* = 0.045; Table 4, Panel B); all remaining language, executive, visuospatial, and multidomain measures yielded non-significant adjusted estimates. Model-level adjusted estimates are also summarized in Supplementary Table S2.

As shown in Table 4, Panel A, selected neurocognitive tests showed within-group changes in both groups, but these findings require cautious interpretation because the neurocognitive analyses were exploratory, the study was neither designed nor powered to establish cognitive efficacy, and nonprimary endpoints were interpreted descriptively. When the family of nonprimary endpoints was assessed with FDR control, no secondary or exploratory result remained significant; the nominal adjusted signal for the Stick Construction and Visual Recognition Test attenuated to q = 0.484 (Supplementary Table S3, Panel A).

### 3.5. Prespecified Propensity Score-Based Sensitivity Analysis for the Primary Outcome

The prespecified stabilized IPTW ATE sensitivity analysis for the primary outcome was consistent with the main adjusted model and did not demonstrate adjusted superiority of RAT. Covariate-balance diagnostics are presented in Supplementary Table S4, Panel A, and the weighted analysis of covariance (ANCOVA) estimates are shown in Supplementary Table S4, Panel B. In the IPTW-weighted ANCOVA, the estimated treatment effect for FMA-UE motor was β = 2.17 (95% CI, −3.63 to 7.98; *p* = 0.464), reinforcing the view that the unadjusted FMA-UE difference reflected a descriptive signal that attenuated once the measured baseline imbalances were addressed. Considered together, the progression from the crude mean change difference (6.79 points) to the covariate-adjusted estimate (4.39 points) and then to the weighted estimate (2.17 points) is compatible with confounding by indication, in which patients with worse baseline motor severity were more likely to receive RAT. In the multiple-imputation sensitivity analysis, the pooled adjusted treatment effect for week-4 FMA-UE motor was 4.52 points (95% CI, −1.91 to 10.94; *p* = 0.168), again indicating no adjusted superiority of RAT (Supplementary Table S3, Panel B).

### 3.6. Responder Analyses According to MCID or Benchmark Thresholds

Clinically meaningful change for the primary endpoint was anchored to a single prespecified FMA-UE threshold of 12.4 points. In raw complete-case analyses, 14 out of 33 RAT patients (42.4%) and 8 out of 32 COT patients (25.0%) reached this threshold (*p* = 0.191), with an adjusted odds ratio of 1.52 (95% CI, 0.45 to 5.14; *p* = 0.505) (Table 5). Under the prespecified stabilized IPTW ATE sensitivity analysis, weighted responder rates were similar between groups (32.1% in RAT vs. 31.6% in COT; Table 5; Supplementary Table S4, Panel C). Supportive exploratory responder analyses for hand grip strength, K-MBI, FIM, and the Boston Naming Test likewise showed no significant adjusted between-group differences and should be interpreted descriptively rather than confirmatorily (Table 5).

## 4. Discussion

The central finding of this study is that end-effector RAT did not demonstrate adjusted superiority over dose-matched COT for upper-limb motor recovery in subacute stroke. Table 3, Panel A shows that both groups improved over the 4-week rehabilitation period, whereas Table 3, Panel B shows that the adjusted between-group difference for the primary FMA-UE motor endpoint was not significant. The prespecified stabilized IPTW sensitivity analysis yielded a concordant non-significant estimate. The larger unadjusted FMA-UE motor gain in the RAT group should therefore be interpreted as a descriptive impairment-level signal rather than as evidence of comparative efficacy. This distinction matters because the study was conducted under routine clinical allocation rather than randomized assignment; adjusted and propensity-weighted analyses provide a more appropriate basis for interpretation than raw change-score comparisons.

Although the between-group age difference did not reach statistical significance, the COT group was numerically older than the RAT group (67.66 vs. 60.15 years). This difference may still be clinically relevant in a rehabilitation context, because older stroke survivors may have lower physiological reserve, greater comorbidity or frailty burden, reduced endurance, and slower adaptation to high-dose task practice. The older age profile of the COT group may therefore have contributed to more modest functional gains or less efficient translation of impairment-level recovery into ADL performance. Because the present sample was not powered to test treatment-by-age interactions, this interpretation should be considered hypothesis-generating.

Our results are consistent with the broader robotic rehabilitation literature. Systematic reviews and meta-analyses generally support the potential of RAT to improve upper-limb impairment, activities of daily living, and arm strength after stroke while emphasizing substantial heterogeneity across device type, stroke phase, intervention dose, comparator intensity, and outcome selection [11,12,13,14]. When robotic therapy is compared with low-intensity or usual-care comparators, larger effects are often observed; when RAT is tested against active, dose-matched therapist-delivered rehabilitation, clear superiority is harder to demonstrate [11,12,13,14]. Our findings align with this more conservative interpretation: in a pragmatic dose-matched comparison, RAT was feasible and associated with improvement but did not yield adjusted superiority over COT.

An important interpretive point is that the present study did not compare robot-only treatment with conventional occupational therapy alone. The RAT group received a combined intervention of 30 min of robot-assisted training followed by 30 min of conventional occupational therapy, whereas the comparison group received 60 min of COT alone. The contrast is therefore best interpreted as RAT-plus-COT versus COT, not RAT versus COT in isolation. Any observed difference may reflect the specific contribution of the robotic component, the sequencing of robotic practice followed by therapist-guided task-oriented practice, or the interaction between these two components, rather than the independent effect of the robot itself. The intervention tested here is more accurately described as a robot-assisted rehabilitation program embedded within comprehensive occupational therapy. This framing is consistent with prior studies in which robotic training was paired with additional functional or ADL-oriented practice to enhance transfer from impairment-level gains to everyday upper-limb use [22].

Our results also support the view that RAT is best considered an intensity-enhancing adjunct within comprehensive rehabilitation rather than a replacement for therapist-led therapy. Earlier Korean and international studies have likewise reported improvement after robotic upper-limb training, but between-group superiority over conventional therapy has been inconsistent, particularly over short intervention periods [22,23,29,30,31]. Differences in device design and training target may further shape treatment response: distal-emphasized robotic training, proximal-emphasized training, end-effector systems, exoskeleton systems, and combined RAT-plus-ADL approaches may not be clinically interchangeable [9,22,30]. In this context, the end-effector platform used here may be particularly suited to delivering repetitive, feedback-enriched reaching practice, while COT may retain advantages for contextualized task training, compensatory strategy use, and direct ADL integration.

The type of robotic system used may also have influenced the pattern of recovery observed in this study. The Rebless Planar is a planar end-effector device well-suited to repetitive, feedback-enriched reaching practice—particularly for proximal transport and trajectory control. Compared with distal-focused systems or exoskeleton-based platforms, however, a planar end-effector approach may provide less direct training of hand shaping, grasp-release dexterity, fine object manipulation, and distal coordination. These distal components are central to independent ADL performance, especially in older patients with reduced reserve for spontaneous transfer from proximal reaching practice to functional hand use. This device-specific consideration may partly explain why impairment-level motor change was more apparent than activity-level improvement in the present study.

The clinical interpretation of the FMA-UE findings should remain measured. The Fugl–Meyer Assessment is a sensitive impairment-level measure for post-stroke motor recovery [32], and the prespecified 12.4-point FMA-UE threshold was selected to limit interpretive inflation from multiple published MCID estimates and to reflect values applicable to patients with moderate-to-severe upper-limb paresis [15,25,33]. In Table 3, Panel A, the mean FMA-UE motor gain in the RAT+COT group exceeded this threshold whereas the COT mean did not, and the raw responder proportion was numerically higher in RAT (Table 5). However, Table 3, Panel B and the IPTW analysis show that this pattern was not confirmed by adjusted regression or propensity-weighted estimation. The FMA-UE result therefore signals a clinically relevant pattern worthy of prospective testing, but it does not establish a confirmed incremental benefit of RAT over dose-matched COT.

The discrepancy between the FMA-UE signal and the more limited between-group differences in MFT, grip strength, K-MBI, and FIM is also informative. Table 3, Panel A shows within-group gains across these motor and functional measures, whereas Table 3, Panel B shows that adjusted between-group effects were not significant. RAT may preferentially influence early impairment-level motor control before such gains translate into broader hand function, ADL performance, or global independence. Repetitive, high-dose, kinematically guided practice is consistent with principles of motor learning and experience-dependent neuroplasticity [2,7,8,11,12,24,34]. Conversion of impairment-level gains into functional independence, however, likely requires sufficient time, task specificity, distal hand engagement, and structured practice in real-world activities. The 4-week observation window in this study should be interpreted as a pragmatic early-response interval rather than as a full representation of subacute stroke recovery. This timepoint matched the institution’s routine inpatient reassessment schedule and is broadly consistent with prior robotic rehabilitation studies that have used 3- to 4-week treatment blocks in subacute stroke [2,21,22,23]. Stroke recovery—particularly activity-level and participation-level recovery in older adults—can extend well beyond the first month, and the subacute neuroplastic window is not confined to 4 weeks. The present results are therefore best understood as estimates of short-term change during an early rehabilitation phase, whereas longer follow-up would be required to determine the durability of motor gains and their translation into ADL performance, participation, and quality of life.

The exploratory neurocognitive findings should be interpreted with similar caution. Cognitive impairment after stroke is common and clinically relevant because it can influence rehabilitation engagement, motor learning, functional recovery, and long-term independence [4,5,6]. Robotic rehabilitation may provide an enriched therapeutic environment through visual feedback, repetitive task engagement, attentional demands, and performance-based progression [16]. The within-group changes shown in Table 4, Panel A should not be interpreted as cognitive efficacy, and the between-group estimates in Table 4, Panel B identified only one nominal adjusted signal. The exploratory neurocognitive findings can also be considered within a cognitive stimulation framework: planar robotic training provides continuous visual feedback, repeated target-oriented movement, and ongoing requirements for visuomotor monitoring and error correction. Such training may engage spatial attention, target localization, online trajectory adjustment, and visuospatial-constructional processing more than non-visual or less feedback-rich practice. It is therefore conceivable that visuospatial tasks could be particularly sensitive to feedback-based flat-surface training. Because the nominal visuospatial finding did not survive FDR correction (Supplementary Table S3, Panel A), however, this interpretation should be regarded only as a mechanistic hypothesis for future prospective studies. These results should not be read as evidence that RAT improves cognition; rather, they suggest that visuospatial, executive, language, and multidomain cognitive measures may merit incorporation into future prospective robotic rehabilitation studies as structured exploratory or mechanistic outcomes.

A strength of this study is its real-world inpatient context. Many rehabilitation trials exclude patients with cognitive impairment, comorbidity, advanced age, or clinical complexity, limiting generalizability to routine stroke rehabilitation practice [17]. Our cohort reflects the type of population in which decisions about RAT versus COT alone are actually made. The study also used a dose-matched comparator, a single prespecified primary motor endpoint, covariate-adjusted analyses, a prespecified propensity-based sensitivity analysis, responder analyses anchored to a clinically interpretable FMA-UE threshold, and supportive handling of multiplicity and missing data. These features do not eliminate bias, but they strengthen the transparency and clinical interpretability of the findings.

Several methodological considerations should be acknowledged without diminishing the pragmatic value of the dataset. First, the retrospective non-randomized design leaves the possibility of selection bias and confounding by indication, particularly because the RAT group had worse baseline upper-limb motor status. Second, although regression adjustment and stabilized IPTW addressed measured covariates and improved balance for the primary propensity-score model variables (Supplementary Table S4, Panel A), they could not account for variables that were not available in model-ready form. Clinically relevant confounders such as lesion location and size, spatial neglect, aphasia severity, depression or mood status, nutritional status, polypharmacy, pre-stroke physical activity level, relevant medication exposure, and concurrent non-study therapies were not consistently available [35,36,37,38,39,40,41]. These factors may have influenced both treatment allocation and recovery trajectories. For example, larger or strategically located lesions, neglect, or more severe aphasia could reduce task participation and apparent responsiveness to training, whereas malnutrition, polypharmacy, and lower premorbid physical activity may indicate reduced physiological reserve, lower endurance, or greater medical complexity during rehabilitation. Residual confounding therefore remains a key limitation of the present non-randomized analysis. Third, outcome-specific complete-case analysis may have introduced bias because week-4 follow-up missingness was more frequent in COT and involved patients with better baseline motor and functional status (Supplementary Table S1), potentially making unadjusted comparisons appear more favorable to RAT. The multiple-imputation analysis for the primary endpoint was concordant with the complete-case result (Supplementary Table S3, Panel B), but it relied on a missing-at-random assumption and could not fully remove uncertainty. Finally, the follow-up period was short, and the neurocognitive analyses were exploratory and underpowered for efficacy inference. These issues limit causal interpretation, but they also clarify the main message: RAT was feasible and associated with clinically interesting motor improvement in routine practice, while adjusted superiority over dose-matched COT was not demonstrated.

Future studies should build on these findings using prospective designs that preserve clinical generalizability while improving causal control. Randomized or carefully stratified pragmatic trials should balance baseline motor severity, standardize treatment fidelity monitoring, prespecify missing-data strategies, and capture lesion characteristics, aphasia, neglect, mood, medication exposure, concurrent therapy dose, frailty, and cognitive reserve. Longer follow-up is needed to determine whether early FMA-UE gains translate into hand function, ADL performance, participation, and quality of life [15,25,33,35,36,37,38]. Future work should also examine whether RAT is most effective when paired with structured ADL-oriented practice, whether proximal and distal robotic targets should be combined or sequenced, and which cognitive or clinical profiles predict engagement and response [39,40,41,42,43]. Taken together, the present study supports RAT as a feasible component of comprehensive subacute stroke rehabilitation and provides a clinically relevant signal for future investigation, but it does not establish adjusted comparative superiority over dose-matched COT.

## 5. Conclusions

In this real-world retrospective non-randomized cohort of patients with subacute stroke, end-effector RAT did not demonstrate adjusted superiority over dose-matched COT for upper-limb motor recovery, and the prespecified stabilized IPTW sensitivity analysis was concordant. The larger unadjusted FMA-UE gain in the RAT group should therefore be interpreted cautiously as a descriptive impairment-level signal only. The exploratory neurocognitive results should likewise be regarded as hypothesis-generating rather than as evidence of cognitive benefit. Because the study was retrospective, baseline imbalance was substantial, potentially important confounders were incompletely captured, and missing data were not negligible, the findings should not be extended to causal claims of comparative efficacy. RAT may be considered a feasible adjunct within comprehensive stroke rehabilitation; because the intervention contrast in this study was effectively RAT-plus-COT versus COT, the findings should not be interpreted as demonstrating the independent effect of robot-only treatment.

## Figures and Tables

**Table 1:** Extent and pattern of missing outcome data in the eligible cohort.

Category	Total	COT	RAT	Notes
Eligible after clinical screening	92	52	40	After exclusion of 44 patients for predefined clinical criteria
No baseline assessment for any prespecified outcome	13	8	5	Excluded before outcome-specific complete-case analyses
Baseline data available for at least one prespecified outcome	79	44	35	Used for missing-data pattern assessment
No 4-week follow-up for any prespecified outcome	14	12	2	Predominantly early discharge or early treatment termination
Analytic complete-case cohort	65	32	33	Included in the main outcome analyses

Abbreviations: RAT, robot-assisted therapy; COT, conventional occupational therapy.

**Table 2:** Baseline demographic, clinical, motor, functional, and neurocognitive characteristics of the study population.

Variable	RAT (*n* = 33)	COT (*n* = 32)	*p*-Value
Age, years	60.15 ± 18.07	67.66 ± 15.15	0.074
Height, cm	163.82 ± 8.92	163.50 ± 9.48	0.890
Weight, kg	70.24 ± 26.00	62.59 ± 11.96	0.133
Onset-to-baseline duration, days	44.06 ± 77.44	27.31 ± 24.19	0.244
Sex			1.000
Male, n (%)	18 (54.5)	17 (53.1)	
Female, n (%)	15 (45.5)	15 (46.9)	
Stroke type			0.450
Hemorrhagic, n (%)	15 (45.5)	11 (34.4)	
Ischemic, n (%)	18 (54.5)	21 (65.6)	
Hemiplegic side			0.460
Right, n (%)	19 (57.6)	15 (46.9)	
Left, n (%)	14 (42.4)	17 (53.1)	
MMSE	19.52 ± 9.78	18.59 ± 9.19	0.697
FMA-UE motor	18.18 ± 20.45	36.06 ± 27.14	**0.004**
FMA total	64.03 ± 27.55	85.25 ± 34.50	**0.008**
Hand grip strength, lb	1.58 ± 4.83	19.78 ± 24.34	**<0.001**
MFT	6.94 ± 8.30	14.62 ± 12.17	**0.004**
K-MBI	29.27 ± 22.72	30.75 ± 25.97	0.808
FIM	59.03 ± 23.49	60.25 ± 23.67	0.836
Boston Naming Test	8.58 ± 5.01	8.31 ± 4.79	0.829
Language Comprehension and Repetition Test	15.48 ± 5.72	15.00 ± 5.81	0.736
Right–Left Orientation Test	2.24 ± 1.17	2.22 ± 1.07	0.932
Stick Construction and Visual Recognition Test	21.03 ± 7.29	18.92 ± 8.82	0.299
Fist-Edge-Palm Test	1.73 ± 1.26	1.75 ± 1.30	0.943
Construction Test	7.33 ± 3.54	7.25 ± 3.84	0.928
Stroop Test: Word	41.97 ± 29.28	36.53 ± 28.24	0.449
Stroop Test: Color	30.85 ± 26.19	24.47 ± 24.82	0.317
Stroop Test: Color–Word	18.70 ± 20.21	15.50 ± 18.45	0.508
Clock Drawing Test	1.58 ± 1.06	1.50 ± 1.30	0.798

Data are presented as mean ± SD or n (%), as appropriate. *p* values were obtained using Welch’s *t*-test for continuous variables and Fisher’s exact test for categorical variables. Bold *p* values indicate statistical significance at *p* < 0.05. Abbreviations: RAT, robot-assisted therapy; COT, conventional occupational therapy; SD, standard deviation; MMSE, Mini-Mental State Examination; FMA, Fugl–Meyer Assessment; FMA-UE, Fugl–Meyer Assessment–Upper Extremity; MFT, Manual Function Test; K-MBI, Korean Modified Barthel Index; FIM, Functional Independence Measure.

**Table 3:** Four-week changes in primary and secondary motor and functional outcomes. (**A**). Within-group baseline-to-week-4 changes. (**B**). Between-group effect estimates.

(A)						
Outcome	RAT Baseline	RAT 4 Weeks	RAT Δ (*p*)	COT Baseline	COT 4 Weeks	COT Δ (*p*)
FMA-UE motor	18.18 ± 20.45	32.88 ± 22.55	14.70 ± 15.53 (<0.001)	36.06 ± 27.14	43.97 ± 24.78	7.91 ± 9.42 (<0.001)
FMA total	64.03 ± 27.55	81.82 ± 27.50	17.79 ± 21.26 (<0.001)	85.25 ± 34.50	95.50 ± 32.66	10.25 ± 10.00 (<0.001)
Hand grip strength, lb	1.58 ± 4.83	9.61 ± 13.38	8.03 ± 12.62 (<0.001)	19.78 ± 24.34	23.12 ± 25.82	3.34 ± 7.32 (0.015)
MFT	6.94 ± 8.30	13.24 ± 9.60	6.30 ± 7.18 (<0.001)	14.62 ± 12.17	19.41 ± 10.65	4.78 ± 5.53 (<0.001)
K-MBI	29.27 ± 22.72	52.36 ± 25.22	23.09 ± 17.81 (<0.001)	30.75 ± 25.97	47.91 ± 29.86	17.16 ± 12.92 (<0.001)
FIM	59.03 ± 23.49	78.45 ± 21.63	19.42 ± 11.39 (<0.001)	60.25 ± 23.67	74.03 ± 30.79	13.78 ± 13.38 (<0.001)
(**B**)						
Outcome		Unadjusted mean difference (95% CI; *p* ᵃ)			Adjusted mean difference (95% CI; *p* ᵇ)	
FMA-UE motor		6.79 (0.42, 13.16) (0.037)			4.39 (−2.43, 11.21) (0.203)	
FMA total		7.54 (−0.72, 15.79) (0.073)			4.34 (−4.24, 12.91) (0.315)	
Hand grip strength, lb		4.69 (−0.43, 9.80) (0.072)			4.12 (−2.04, 10.28) (0.186)	
MFT		1.52 (−1.65, 4.70) (0.342)			−0.11 (−3.52, 3.30) (0.948)	
K-MBI		5.93 (−1.77, 13.64) (0.129)			4.04 (−3.22, 11.30) (0.270)	
FIM		5.64 (−0.53, 11.82) (0.072)			4.45 (−1.21, 10.10) (0.121)	

Values are mean ± SD. Δ = week 4 − baseline; the *p* value in parentheses in Panel A is the within-group *p* value from paired *t*-tests. ᵃ Unadjusted between-group comparison from Welch’s *t*-test on change scores. ᵇ Adjusted mean difference from multivariable linear regression with the week-4 score as the dependent variable and treatment group, baseline score, age, sex, stroke type, onset duration, and baseline MMSE as covariates. The primary outcome (FMA-UE motor) is shown in bold. Abbreviations: RAT, robot-assisted therapy; COT, conventional occupational therapy; SD, standard deviation; CI, confidence interval; FMA, Fugl–Meyer Assessment; FMA-UE, Fugl–Meyer Assessment–Upper Extremity; MFT, Manual Function Test; K-MBI, Korean Modified Barthel Index; FIM, Functional Independence Measure; Adj., adjusted.

**Table 4:** Four-week changes in exploratory neurocognitive outcomes by cognitive domain. (A). Within-group baseline-to-week-4 changes. (B). Between-group effect estimates.

(A)						
Outcome	RAT Baseline	RAT 4 Weeks	RAT Δ (*p*)	COT Baseline	COT 4 Weeks	COT Δ (*p*)
Language						
Boston Naming Test	8.58 ± 5.01	10.85 ± 3.85	2.27 ± 2.85 (<0.001)	8.31 ± 4.79	9.78 ± 4.43	1.47 ± 2.87 (0.007)
Language Comp. and Rep. Test	15.48 ± 5.72	16.42 ± 4.82	0.94 ± 2.60 (0.046)	15.00 ± 5.81	16.69 ± 4.75	1.69 ± 2.64 (0.001)
Visuospatial and Construction						
Right–Left Orientation Test	2.24 ± 1.17	2.55 ± 0.83	0.30 ± 0.95 (0.077)	2.22 ± 1.07	2.47 ± 0.95	0.25 ± 0.72 (0.058)
Stick Const. and Visual Rec. Test	21.03 ± 7.29	23.95 ± 5.15	2.92 ± 4.46 (<0.001)	18.92 ± 8.82	20.80 ± 7.75	1.88 ± 4.55 (0.026)
Executive Function						
Stroop Test: Word	41.97 ± 29.28	55.36 ± 30.87	13.39 ± 15.36 (<0.001)	36.53 ± 28.24	47.62 ± 31.14	11.09 ± 13.17 (<0.001)
Stroop Test: Color	30.85 ± 26.19	39.58 ± 26.66	8.73 ± 11.12 (<0.001)	24.47 ± 24.82	33.78 ± 28.91	9.31 ± 11.60 (<0.001)
Stroop Test: Color–Word	18.70 ± 20.21	26.73 ± 22.04	8.03 ± 10.99 (<0.001)	15.50 ± 18.45	22.09 ± 23.15	6.59 ± 9.99 (<0.001)
Multidomain						
Clock Drawing Test	1.58 ± 1.06	2.21 ± 0.93	0.64 ± 0.93 (<0.001)	1.50 ± 1.30	1.81 ± 1.26	0.31 ± 1.15 (0.134)
(**B**)						
Outcome	Unadjusted mean difference (95% CI; *p* ᵃ)				Adjusted mean difference (95% CI; *p* ᵇ)	
Language						
Boston Naming Test	0.80 (−0.62, 2.22) (0.262)				0.72 (−0.32, 1.77) (0.169)	
Language Comp. and Rep. Test	−0.75 (−2.05, 0.55) (0.254)				−0.53 (−1.62, 0.55) (0.330)	
Visuospatial and Construction						
Right–Left Orientation Test	0.05 (−0.36, 0.47) (0.800)				0.09 (−0.21, 0.39) (0.544)	
Stick Const. and Visual Rec. Test	1.05 (−1.18, 3.28) (0.351)				1.98 (0.04, 3.91) (0.045)	
Executive Function						
Stroop Test: Word	2.30 (−4.79, 9.39) (0.519)				2.04 (−4.13, 8.20) (0.511)	
Stroop Test: Color	−0.59 (−6.22, 5.05) (0.836)				−0.33 (−5.73, 5.07) (0.904)	
Stroop Test: Color–Word	1.44 (−3.77, 6.64) (0.583)				0.32 (−4.35, 4.99) (0.890)	
Multidomain						
Clock Drawing Test	0.32 (−0.20, 0.84) (0.217)				0.25 (−0.12, 0.62) (0.179)	

Neurocognitive outcomes were grouped a priori into language, visuospatial/constructional, executive, and multidomain measures. Δ = week 4 − baseline; the *p* value in parentheses in Panel A is the within-group *p* value from paired *t*-tests. ᵃ Unadjusted between-group comparison from Welch’s *t*-test on change scores. ᵇ Adjusted mean difference from multivariable linear regression with the week-4 score as the dependent variable and treatment group, baseline score, age, sex, stroke type, onset duration, and baseline MMSE as covariates. These analyses were exploratory and should be interpreted as hypothesis-generating. Abbreviations: RAT, robot-assisted therapy; COT, conventional occupational therapy; SD, standard deviation; CI, confidence interval; MMSE, Mini-Mental State Examination; Comp., comprehension; Rep., repetition; Const., construction; Rec., recognition; CI, confidence interval; Adj., adjusted.

**Table 5:** Responder analyses according to clinically meaningful change thresholds.

Outcome	Threshold	COT Responders	RAT Responders	*p*-Value	Adjusted OR (95% CI)	Adj. OR *p*
FMA-UE motor (complete-case)	≥12.4	8/32 (25.0)	14/33 (42.4)	0.191	1.52 (0.45, 5.14)	0.505
FMA-UE motor (stabilized IPTW ATE-weighted)	≥12.4	31.6%	32.1%	—	—	—
Hand grip strength, lb	11.02	4/32 (12.5)	9/33 (27.3)	0.215	2.60 (0.46, 14.55)	0.278
K-MBI	9.25	23/32 (71.9)	26/33 (78.8)	0.574	0.86 (0.21, 3.55)	0.835
FIM	22	10/32 (31.2)	14/33 (42.4)	0.443	1.59 (0.48, 5.25)	0.448
Boston Naming Test	3.3	6/32 (18.8)	6/33 (18.2)	1.000	1.71 (0.18, 16.33)	0.643

Responder analyses were based on published MCID thresholds or benchmark definitions. Between-group *p* values were obtained using Fisher’s exact test. Adjusted odds ratios (ORs) were estimated using multivariable logistic regression (covariates: treatment group, baseline score, age, sex, stroke type, onset duration, baseline MMSE). For FMA-UE motor, stabilized IPTW ATE-weighted responder rates were additionally calculated as the prespecified propensity-score-based sensitivity analysis. The Boston Naming Test threshold represents a benchmark of clinically important change. Abbreviations: RAT, robot-assisted therapy; COT, conventional occupational therapy; OR, odds ratio; CI, confidence interval; MMSE, Mini-Mental State Examination; FMA-UE, Fugl–Meyer Assessment–Upper Extremity; MCID, minimal clinically important difference; IPTW, inverse probability of treatment weighting; ATE, average treatment effect; K-MBI, Korean Modified Barthel Index; FIM, functional independence measure; Adj., adjusted.

## Data Availability

The data presented in this study are available on reasonable request from the corresponding author. The data are not publicly available due to privacy and ethical restrictions related to patient-level clinical information.

## Supplementary Materials

The following supporting information can be downloaded at: https://www.mdpi.com/article/10.3390/jcm15093512/s1, Table S1: Baseline comparison of the complete-case cohort versus patients with missing week-4 follow-up among the baseline-evaluable cohort; Table S2: Multivariable regression analyses of the association between treatment group and four-week outcomes; Table S3: False discovery rate-adjusted *p* values for nonprimary endpoints and multiple-imputation sensitivity analysis for the primary outcome; Table S4: Propensity score balance diagnostics and prespecified stabilized IPTW sensitivity analyses for the primary outcome.

## Abbreviations

The following abbreviations are used in this manuscript:

ADL	Activities of daily living
AI	Artificial intelligence
ANCOVA	Analysis of covariance
ATE	Average treatment effect
BNT	Boston Naming Test
CI	Confidence interval
COT	Conventional occupational therapy
FDR	False discovery rate
FIM	Functional independence measure
FMA	Fugl-Meyer Assessment
FMA-UE	Fugl–Meyer Assessment—Upper Extremity
IPTW	Inverse probability of treatment weighting
K-MBI	Korean Modified Barthel Index
MCID	minimal clinically important difference
MFT	Manual Function Test
MMSE	Mini-Mental State Examination
OR	Odds ratio
PS	Propensity score
RAT	Robot-assisted therapy
R^2^	Coefficient of determination
SD	Standard deviation
SE	Standard error
SMD	Standardized mean difference
STROBE	Strengthening the Reporting of Observational Studies in Epidemiology
TBI	Traumatic brain injury
