# Supplementary material for: Motor Outcomes of Robot-Assisted Versus Conventional Occupational Therapy for Upper-Limb Recovery in Subacute Stroke: A Retrospective Cohort Study with Exploratory Neurocognitive Outcomes
Source: J Clin Med. 2026 May 4;15(9):3512. doi: 10.3390/jcm15093512 (PMC13163380; doi:10.3390/jcm15093512)
Supplement: Supplementary file 1 [file jcm-15-03512-s001.zip › Supplementary Tables 1.pdf]

Supplementary Table S1. Baseline comparison between the complete-case cohort and patients with missing week-4 follow-up among the baseline-evaluable cohort.

Variable	Complete-case cohort (n = 65)	Missing week-4 follow-up (n = 14)	p-Value
Age, years	63.85 ± 16.99	65.93 ± 13.74	0.628
Male sex, n (%)	34/65 (52.3)	9/14 (64.3)	0.557
Ischemic stroke, n (%)	39/65 (60.0)	10/14 (71.4)	0.549
RAT group, n (%)	33/65 (50.8)	2/14 (14.3)	0.017
Onset-to-baseline duration, days	35.82 ± 57.90	19.43 ± 21.41	0.080
Baseline MMSE	19.06 ± 9.43	20.23 ± 8.94	0.675
Baseline FMA-UE motor	26.98 ± 25.44	50.08 ± 26.58	0.014
Baseline K-MBI	30.00 ± 24.19	51.21 ± 29.54	0.022
Baseline FIM	59.63 ± 23.40	80.79 ± 30.53	0.026

Data are presented as mean ± SD or n (%). Continuous variables were compared with Welch's t-test and categorical variables with Fisher's exact test. Bold p values indicate $p < 0.05$.

Abbreviations: RAT, robot-assisted therapy; MMSE, Mini-Mental State Examination; FMA-UE, Fugl–Meyer Assessment—Upper Extremity; K-MBI, Korean Modified Barthel Index; FIM, Functional Independence Measure.
