# Supplementary material for: Motor Outcomes of Robot-Assisted Versus Conventional Occupational Therapy for Upper-Limb Recovery in Subacute Stroke: A Retrospective Cohort Study with Exploratory Neurocognitive Outcomes
Source: J Clin Med. 2026 May 4;15(9):3512. doi: 10.3390/jcm15093512 (PMC13163380; doi:10.3390/jcm15093512)
Supplement: Supplementary file 1 [file jcm-15-03512-s001.zip › Supplementary Tables 2.pdf]

Supplementary Table S2. Multivariable regression analyses of the association between treatment group and four-week outcomes.

| **Outcome** | **n** | **Adjusted β (95% CI)** | **p-Value** | **$R^2$** |
|---|---|---|---|---|
| FMA-UE motor | 65 | 4.39 (−2.43, 11.21) | 0.203 | 0.765 |
| FMA total | 65 | 4.34 (−4.24, 12.91) | 0.315 | 0.766 |
| Hand grip strength, lb | 65 | 4.12 (−2.04, 10.28) | 0.186 | 0.794 |
| MFT | 65 | −0.11 (−3.52, 3.30) | 0.948 | 0.694 |
| FIM | 65 | 4.45 (−1.21, 10.10) | 0.121 | 0.848 |
| K-MBI | 65 | 4.04 (−3.22, 11.30) | 0.270 | 0.768 |
| Boston Naming Test | 65 | 0.72 (−0.32, 1.77) | 0.169 | 0.790 |
| Language Comp. and Rep. Test | 65 | −0.53 (−1.62, 0.55) | 0.330 | 0.826 |
| Right–Left Orientation Test | 65 | 0.09 (−0.21, 0.39) | 0.544 | 0.613 |
| Stick Const. and Visual Rec. Test | 65 | 1.98 (0.04, 3.91) | 0.045 | 0.723 |
| Stroop Test: Word | 65 | 2.04 (−4.13, 8.20) | 0.511 | 0.868 |
| Stroop Test: Color | 65 | −0.33 (−5.73, 5.07) | 0.904 | 0.874 |
| Stroop Test: Color-Word | 65 | 0.32 (−4.35, 4.99) | 0.890 | 0.857 |
| Clock Drawing Test | 65 | 0.25 (−0.12, 0.62) | 0.179 | 0.637 |

Linear regression models were fitted with the week-4 score as the dependent variable. Covariates included treatment group, baseline score, age, sex, stroke type, onset duration, and baseline MMSE. The adjusted β represents the treatment-group coefficient; positive values favor RAT. Bold p values indicate $p < 0.05$.

Abbreviations: β, regression coefficient; CI, confidence interval; $R^2$, coefficient of determination; FMA-UE, Fugl–Meyer Assessment—Upper Extremity; MFT, Manual Function Test; K-MBI, Korean Modified Barthel Index; FIM, Functional Independence Measure; Comp., Comprehension; Rep., Repetition; Const., Construction; Rec., Recognition.
