# Supplementary material for: Motor Outcomes of Robot-Assisted Versus Conventional Occupational Therapy for Upper-Limb Recovery in Subacute Stroke: A Retrospective Cohort Study with Exploratory Neurocognitive Outcomes
Source: J Clin Med. 2026 May 4;15(9):3512. doi: 10.3390/jcm15093512 (PMC13163380; doi:10.3390/jcm15093512)
Supplement: Supplementary file 1 [file jcm-15-03512-s001.zip › Supplementary Tables 3.pdf]

Supplementary Table S3. False discovery rate-adjusted p values for nonprimary endpoints and multiple-imputation sensitivity analysis for the primary outcome.

Panel A. Benjamini-Hochberg false discovery rate adjustment for nonprimary endpoints

**Outcome**	Model p-Value	**BH-FDR q-Value**
FMA total	0.315	0.536
Hand grip strength, lb	0.186	0.484
MFT	0.948	0.948
K-MBI	0.270	0.536
FIM	0.121	0.484
Boston Naming Test	0.169	0.484
Language Comprehension and Repetition Test	0.330	0.536
Right–Left Orientation Test	0.544	0.707
Stick Construction and Visual Recognition Test	0.045	0.484
Stroop Test: Word	0.511	0.707
Stroop Test: Color	0.904	0.948
Stroop Test: Color-Word	0.890	0.948
Clock Drawing Test	0.179	0.484

No nonprimary endpoint remained significant after false discovery rate control. The primary endpoint was not included in this nonprimary endpoint family.

Panel B. Multiple-imputation sensitivity analysis for the primary outcome (FMA-UE motor)

**Model**	**N**	**Adjusted β**	**95% CI**	**p-Value**
Complete-case multivariable ANCOVA	65	4.39	-2.43 to 11.21	0.203
Multiple imputation by chained equations (30 imputations)	79	4.52	-1.91 to 10.94	0.168

The multiple-imputation model included treatment group, baseline FMA-UE motor, age, sex, stroke type, onset duration, baseline MMSE, baseline K-MBI, and baseline FIM. Imputation was performed under a missing-at-random assumption using 30 imputations, and pooled estimates were derived with Rubin's rules.

Abbreviations: BH-FDR, Benjamini-Hochberg false discovery rate; CI, confidence interval; ANCOVA, analysis of covariance; FMA-UE, Fugl–Meyer Assessment—Upper Extremity; MMSE, Mini-Mental State Examination; K-MBI, Korean Modified Barthel Index; FIM, Functional Independence Measure.
