# Supplementary material for: Motor Outcomes of Robot-Assisted Versus Conventional Occupational Therapy for Upper-Limb Recovery in Subacute Stroke: A Retrospective Cohort Study with Exploratory Neurocognitive Outcomes
Source: J Clin Med. 2026 May 4;15(9):3512. doi: 10.3390/jcm15093512 (PMC13163380; doi:10.3390/jcm15093512)
Supplement: Supplementary file 1 [file jcm-15-03512-s001.zip › Supplementary Tables 4.pdf]

Supplementary Table S4. Propensity score balance diagnostics and prespecified stabilized IPTW sensitivity analyses for the primary outcome (FMA-UE motor).

Panel A. Propensity score model and covariate balance

**Variable**	**Raw SMD Before**	**Raw SMD IPTW ATE**	**Raw SMD 1:1 Matched**	**|SMD| Before**	**|SMD| IPTW ATE**	**|SMD| 1:1 Matched**
Age	−0.450	−0.081	0.086	0.450	0.081	0.086
K-MBI baseline	−0.061	−0.033	0.084	0.061	0.033	0.084
MMSE baseline	0.097	−0.052	−0.113	0.097	0.052	0.113
FMA-UE motor baseline	−0.744	0.007	0.026	0.744	0.007	0.026
Sex (male)	0.028	0.153	−0.113	0.028	0.153	0.113
Diagnosis (ischemic)	−0.226	0.003	−0.116	0.226	0.003	0.116
Paretic side (right)	0.214	0.193	0.111	0.214	0.193	0.111

Abbreviations: SMD, standardized mean difference; |SMD|, absolute standardized mean difference; IPTW, inverse probability of treatment weighting; ATE, average treatment effect. Absolute SMD values are shown to aid interpretation; lower values indicate better covariate balance, and |SMD| < 0.10 is commonly used as an indicator of adequate balance.

Panel B. Prespecified stabilized IPTW sensitivity analyses for FMA-UE motor

**Model**	**N**	**Adj. β**	**95% CI Lower**	**95% CI Upper**	**p-Value**	**Note**
Multivariable ANCOVA (reference)	65	4.39	−2.43	11.21	0.203	Post FMA ~ treatment + baseline FMA + covariates
IPTW ATE weighted ANCOVA	65	2.17	−3.63	7.98	0.464	Stabilized IPTW

Abbreviations: PS, propensity score; IPTW, inverse probability of treatment weighting; ATE, average treatment effect; Adj., adjusted.

Panel C. FMA-UE motor responder rates in the complete-case and prespecified stabilized IPTW analyses

**Analysis**	**Threshold**	**COT Rate**	**RAT Rate**
Raw complete-case	FMA change ≥ 12.4	25.0%	42.4%
IPTW ATE weighted	FMA change ≥ 12.4	31.6%	32.1%

Propensity scores were estimated using age, sex, stroke type, paretic side, baseline K-MBI, baseline MMSE, and baseline FMA-UE motor score. The 1:1 matched columns in Panel A are presented only as auxiliary covariate-balance diagnostics; matching was not used as an analytic sensitivity analysis or treatment-effect estimator, and no matched-cohort outcome model was fitted. The prespecified propensity-based analytic sensitivity analysis was the stabilized IPTW ATE weighted ANCOVA shown in Panel B.

Abbreviations: IPTW, inverse probability of treatment weighting; ATE, average treatment effect; PS, propensity score.
